# Small Tumor, Major Complication: A Case of Spontaneous Perinephric Hematoma From a Small Renal Angiomyolipoma

**DOI:** 10.7759/cureus.44761

**Published:** 2023-09-06

**Authors:** Deepak Kumar, Ankur Mittal, Vikas Panwar, Harkirat Talwar, Gurpremjit J Singh

**Affiliations:** 1 Urology, All India Institute of Medical Sciences (AIIMS) Rishikesh, Rishikesh, IND

**Keywords:** wunderlich syndrome, angioembolization, large perinephric hematoma, aneurysm in renal angiomyolipoma, renal angiomyolipoma

## Abstract

A renal angiomyolipoma is a benign kidney tumor composed of muscle, fat, and blood vessels. It is the most common benign kidney tumor, and it affects women more frequently than men. Angiomyolipomas can be small and asymptomatic, or they can be large, presenting with symptoms such as discomfort, hematuria, and hypertension. Occasionally, the rupture of an angiomyolipoma can cause a perinephric hematoma.

This case report discusses a patient who developed a spontaneous large perinephric hematoma alongside a small renal angiomyolipoma. Aneurysm was seen on imaging. Angioembolization was successfully used for treatment. We explore the significance of the presence or absence of an aneurysm in predicting the risk of hemorrhage, particularly in association with small lesions. Angioembolization is an excellent choice for treating angiomyolipomas associated with significant hematomas.

## Introduction

Renal angiomyolipomas are benign kidney tumors that consist of varying proportions of adipose tissue, smooth muscle, and blood vessels. Most of the time, angiomyolipomas develop incidentally, without any associated symptoms. The classic triad of symptoms, flank pain, a palpable mass, and hematuria only, presents in fewer than half of the cases [1,2]. In association with Wunderlich syndrome, spontaneous bleeding can occur in up to 15% of renal angiomyolipomas [3].

Traditionally, the management of renal angiomyolipomas involved nephrectomy, as distinguishing these lesions from renal cell carcinoma was challenging. However, advancements in computed tomography (CT) and ultrasonography have resolved this diagnostic conundrum. Currently, the management of angiomyolipomas is tailored precisely based on their size, propensity for spontaneous hematoma formation, and evidence of bleeding or hematoma [4].

The risk of bleeding is influenced by several factors, including the size of the tumor, the presence of thick-walled arteries, and the emergence of accompanying microaneurysms [5]. Additionally, the likelihood of spontaneous bleeding increases during pregnancy [4]. Spontaneous hematoma is one of the serious complications, seen in up to 10% of cases of angiomyolipoma [6]. This report discusses a case involving a small angiomyolipoma accompanied by a perinephric hematoma. This article was previously presented as a poster at USICON (Annual Conference of the Urological Society of India) on January 25, 2020.

## Case presentation

We report the case of a 55-year-old man with no concurrent conditions who was found to have a renal angiomyolipoma, 3 cm in diameter, during routine follow-up over three months. He experienced right flank pain for 15 days prior to presentation, which radiated to his back. His medical history was unremarkable, with no instances of trauma, fever, or hematuria. Both his heart rate and blood pressure were within normal ranges at the time of presentation. An abdominal examination revealed no abnormalities.

**Figure 1:**
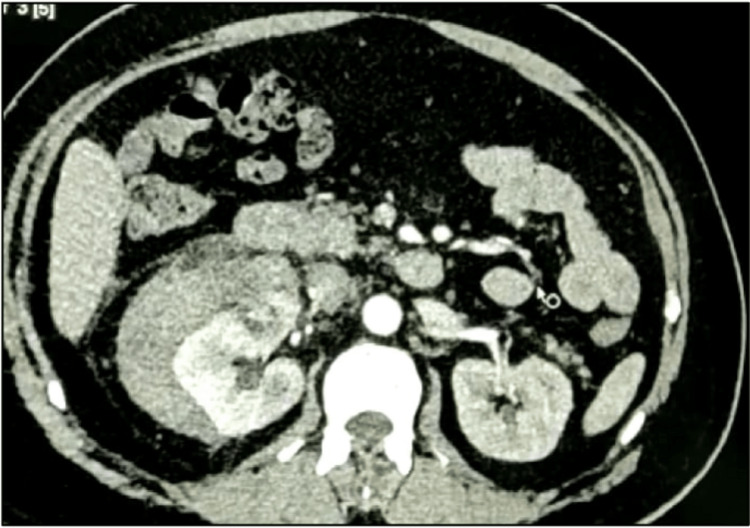
Computed tomogram showing a 2.8 cm angiomyolipoma of the right kidney

**Figure 2:**
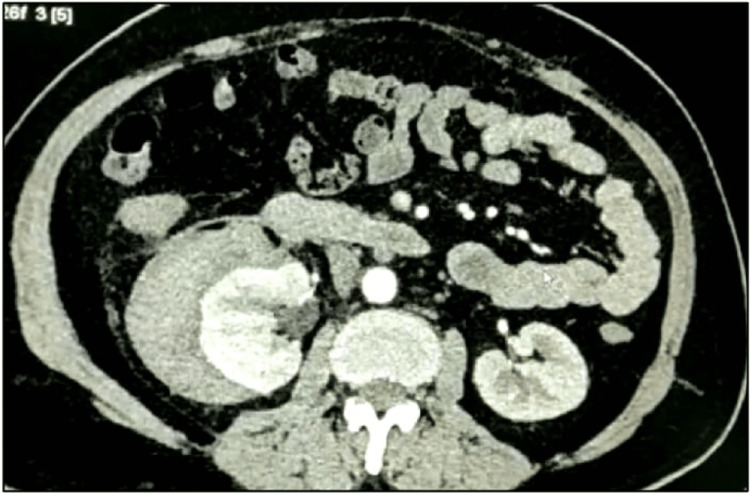
Computed tomogram showing the right kidney compressed medially by hematoma formation

Abdominal ultrasonography revealed a well-defined hyperechoic lesion in the right kidney, suggestive of an angiomyolipoma, with an associated perinephric collection. A subsequent contrast-enhanced CT scan confirmed a 3×2.5 cm angiomyolipoma at the upper pole of the right kidney by the presence of fat and continuity of hematoma from the lesion. and a substantial right perinephric collection measuring approximately 16×10×8 cm (Figure 1 and Figure 2).

**Figure 3:**
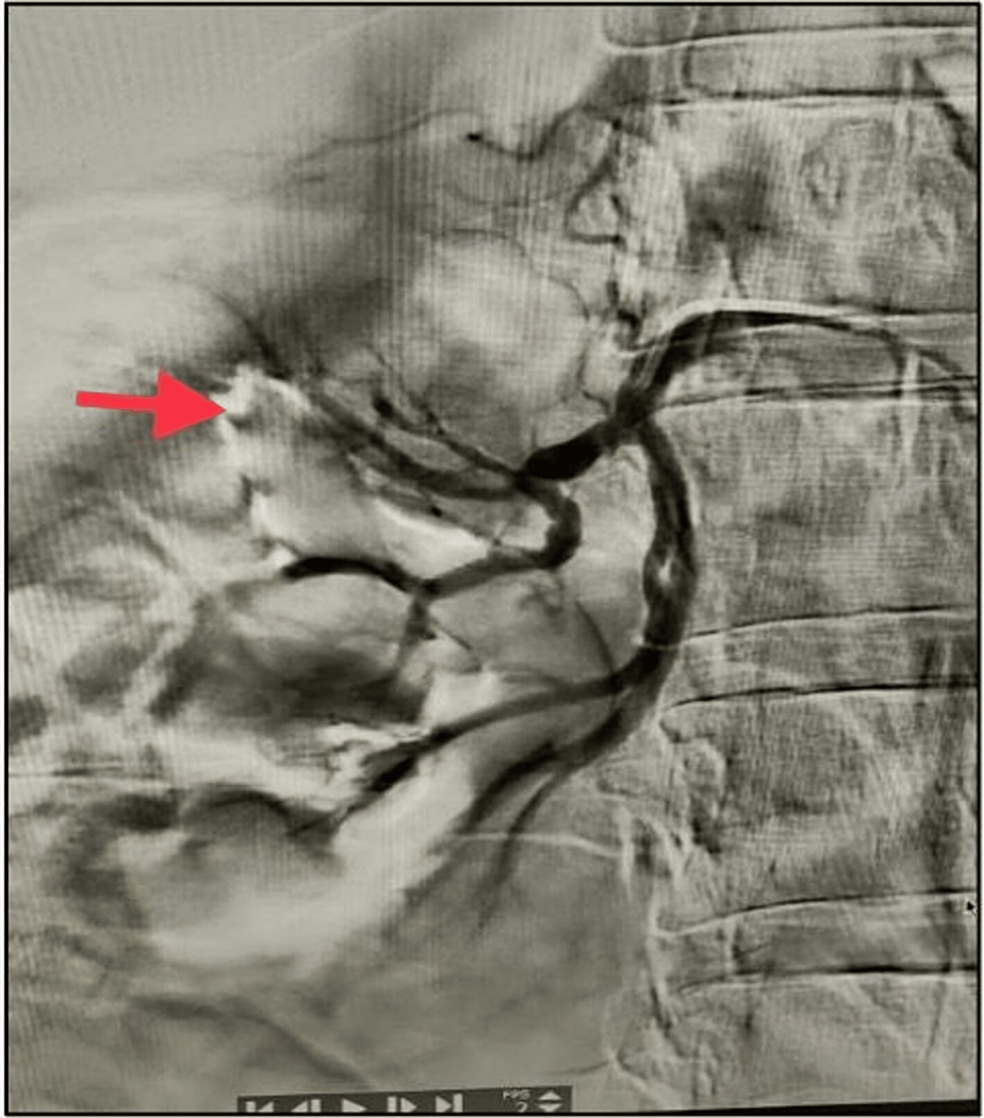
Angiography showing a pseudoaneurysm of the right posterior segmental artery (arrow pointing to pseudoaneurysm site)

**Figure 4:**
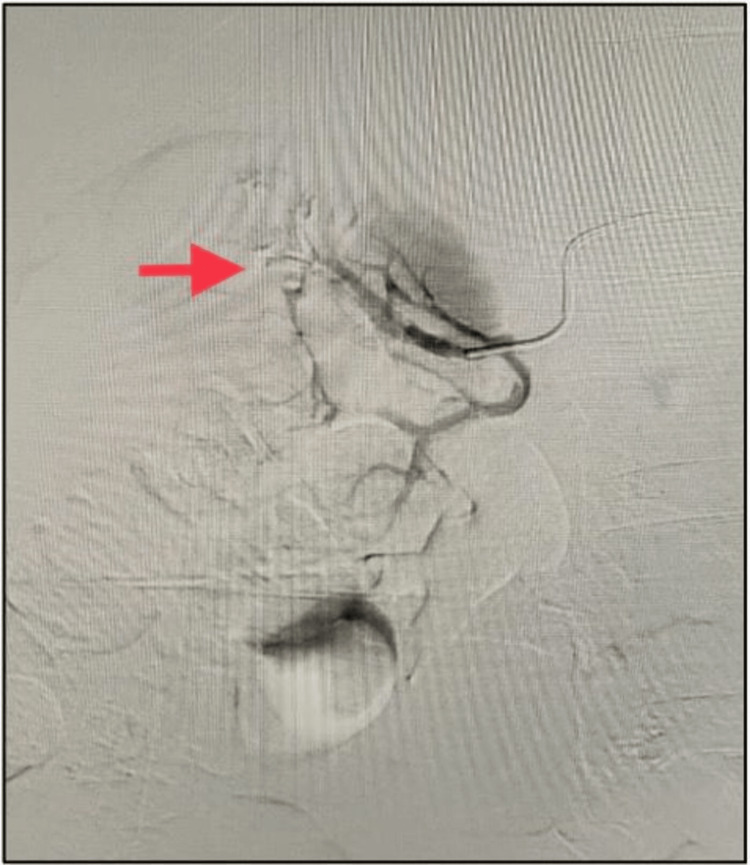
Selective angioembolization of pseudoaneurysm (arrow pointing to pseudoaneurysm site)

The patient's hemoglobin level remained stable. The diagnosis was established as a small right angiomyolipoma with a spontaneous right perinephric hematoma. Owing to the significant perinephric hematoma, angioembolization was planned for the patient. Digital subtraction angiography revealed an aneurysm in the right posterior segmental artery feeding the upper pole (Figure 3). It was selectively embolized using a mixture of lipiodol and n-butyl cyanoacrylate glue (NBCA) (Figure 4).

The procedure was performed efficiently, and the patient was discharged from the hospital 36 hours later in stable condition. A follow-up appointment was scheduled after three months. Subsequent ultrasonography revealed no perinephric collection and a small hyperechoic lesion at the upper pole of the right kidney.

## Discussion

Angiomyolipomas are more likely to develop in females than in males [5]. De Luca et al. reported a female-to-male ratio of 4:1 [4]. Angiomyolipomas typically occur in the fourth and fifth decades of life [7]. They can manifest as a spontaneous condition or due to hereditary predisposition. Most solitary angiomyolipomas occur sporadically, whereas 10-20% of cases demonstrate a genetic predisposition [8].

Hereditary angiomyolipomas are most commonly associated with conditions such as tuberous sclerosis, von Hippel-Lindau disease, and neurofibromatosis type 1. The histologic features of angiomyolipomas were first described by Fischer in 1911, and the term “angiomyolipoma” was coined by Morgan et al. in 1951 [9].

Angiomyolipomas originate from perivascular epithelial cells and are composed of fat, blood vessels, and muscle components. Owing to the presence of fat and blood vessels, angiomyolipomas appear hyperechoic on ultrasonography [10]. On a non-enhanced CT scan, the presence of intratumoral fat (measured at -15 to -20 HU) is diagnostic of angiomyolipoma. A finding of 20 pixels at -20 HU or ≥6 pixels at <-30 HU has a 100% positive predictive value for the diagnosis [11]. Fat-poor angiomyolipomas, which are difficult to diagnose using CT, may be identified using MRI [12].

Most small renal angiolipomas (<4 cm in diameter) are asymptomatic at presentation and can be managed expectantly with repeat imaging every 6 to 12 months to monitor the growth rate [8]. Most angiomyolipomas that bleed are usually >4 cm in diameter. However, the size of the lesion alone may not be a reliable criterion for predicting hemorrhage, as there are multiple other predictive factors. The risk of angiomyolipoma rupture should be based on a comprehensive risk assessment system that includes tumor size, aneurysm formation, pregnancy, coagulopathy, trauma, hormone levels, and comorbidity with tuberous sclerosis complex/lymphangioleiomyomatosis (TSC/LAM). In our case, the presence of an aneurysm was the risk factor for a spontaneous hematoma in the lesion. 

Schieda et al. demonstrated that aneurysm size has a higher statistical value for predicting hemorrhage than angiomyolipoma size [13]. Patients with tuberous sclerosis who have lost the TSC1 and TSC2 genes have a higher risk of hemorrhage due to uncontrolled levels of mTOR (mammalian target of rapamycin) activation. This case report highlights a unique clinical scenario of a patient with no comorbidities, a renal angiomyolipoma <3 cm in diameter, and an unusually large 16 cm hematoma. The patient was successfully managed with angioembolization.

Small angiomyolipomas (<4 cm) also have the risk of spontaneous hemorrhage in 9% of cases [14]. This case illustrates the significance of the presence or absence of an aneurysm in predicting the risk of hemorrhage associated with renal angiomyolipomas, even in cases with smaller lesions. It also underscores the effectiveness of angioembolization as a treatment modality for managing large hematomas.

## Conclusions

The presence or absence of an aneurysm is a critical factor to consider when planning treatment for patients with angiomyolipomas. Even in association with small angiomyolipomas (<4 cm), aneurysms >5 mm in size warrant close monitoring. Angioembolization is the preferred treatment for patients with large perinephric hematomas.
